# The Adelaide Kuru Team in 1957–1959

**DOI:** 10.1098/rstb.2008.4030

**Published:** 2008-11-27

**Authors:** Donald A. Simpson

**Affiliations:** 7A Undelcarra RoadBurnside, SA 5066, Australia

In 1957, Prof. H. N. Robson, then Head of the Department of Medicine in the University of Adelaide, decided to form a small team to study the new disease kuru, recently reported from the highlands of New Guinea by V. Zigas and D. C. Gajdusek. Henry Bennett (Professor of Genetics) remembers that Robson was encouraged to do this by John Eccles and Sydney Sunderland, then great figures in Australian neuroscience (J. H. Bennett 2008, personal communication). Together with F. A. Rhodes (physician, Port Moresby), Robson reconnoitred the kuru area (Bennett *et al.* 1958) and decided to promote more detailed studies of the neurological manifestations of the disease. I was then senior registrar to the Department of Neurosurgery in the Royal Adelaide Hospital. I was released from my ordinary duties to go to Papua New Guinea (PNG) as a member of Robson's team, because it was not possible to get a neurologist to go there at short notice. I went to Okapa together with the late Dr Harry Lander (general physician in Robson's university department) and, from December 1957 to January 1958, we examined a number of victims of kuru, tabulating the neurological findings in 27 cases. We recorded marked ataxia apparently of cerebellar type in all cases. We also examined four cases of alleged recovery from kuru, in whom we found no signs of organic disease; we suspected that these persons had suffered from functional disorders. In many long talks, we discussed kuru with Carleton Gajdusek and Vin Zigas, and with Jack Baker, the very able patrol officer in the area. When we returned to Adelaide, we published our findings in the *Australasian Annals of Medicine* (Simpson *et al.* 1959), stressing the prominence of cerebellar symptomatology. I think that this was our chief neurological contribution; our observations on mental changes and speech impairments were limited by our need to communicate through interpreters. Three autopsies had been done, and the findings were reported by the late Malcolm Fowler, Director of Pathology in the Adelaide Children's Hospital and a very gifted neuropathologist (Fowler & Robertson 1959). In January 1959, I made a second visit to Okapa, accompanied and assisted by my late wife Joanna. I confirmed the fatal course of most or all of the cases seen by me in 1957, and examined seven more cases of kuru. I did not publish anything after this visit; I returned to neurosurgery and never went to PNG again.

I felt highly privileged to see something of kuru in these two short visits and to meet many members of the Fore people in their magically beautiful world. I liked and admired Carleton Gajdusek, and I am proud to write that we have been friends ever since. He gave me much invaluable help in understanding prion diseases, which I acknowledged in a later study of iatrogenic Creutzfeldt–Jakob disease (Simpson *et al.* 1996).
